# Bacteriophages isolated from dairy farm mitigated *Klebsiella pneumoniae*-induced inflammation in bovine mammary epithelial cells cultured *in vitro*

**DOI:** 10.1186/s12917-020-02738-0

**Published:** 2021-01-19

**Authors:** Yuxiang Shi, Wenpeng Zhao, Gang Liu, Tariq Ali, Peng Chen, Yongxia Liu, John P. Kastelic, Bo Han, Jian Gao

**Affiliations:** 1 College of Veterinary Medicine, China Agricultural University, Yuan Ming Yuan West Road No. 2, Haidian District, 100193 Beijing, P. R. China; 2 College of Life Sciences and Food Engineering, Hebei University of Engineering, 056038 Handan, Hebei P.R. China; 3 Center of Microbiology & Biotechnology, Veterinary Research Institute, Peshawar, Pakistan; 4 College of Veterinary Medicine, Shandong Agricultural University, 271018 Taìan, China; 5 Department of Production Animal Health, Faculty of Veterinary Medicine, University of Calgary, T2N 4N1 Calgary, AB Canada

**Keywords:** *Klebsiella pneumoniae*, Bovine mastitis, Bacteriophage, Inflammation, Apoptosis, bMECs

## Abstract

**Background:**

*Klebsiella pneumoniae*, an environmental pathogen causing mastitis in dairy cattle, is often resistant to antibiotics. *K. pneumoniae* was used as the host bacteria to support bacteriophage replication; 2 bacteriophages, CM8-1 and SJT-2 were isolated and considered to have therapeutic potential. In the present study, we determined the ability of these 2 bacteriophages to mitigate cytotoxicity, pathomorphological changes, inflammatory responses and apoptosis induced by *K. pneumoniae* (bacteriophage to *K. pneumoniae* MOI 1:10) in bovine mammary epithelial cells (bMECs) cultured *in vitro*.

**Results:**

Bacteriophages reduced bacterial adhesion and invasion and cytotoxicity (lactate dehydrogenase release). Morphological changes in bMECs, including swelling, shrinkage, necrosis and hematoxylin and eosin staining of cytoplasm, were apparent 4 to 8 h after infection with *K. pneumoniae*, but each bacteriophage significantly suppressed damage and decreased TNF-α and IL-1β concentrations. *K. pneumoniae* enhanced mRNA expression of TLR4, NF-κB, TNF-α, IL-1β, IL-6, IL-8, caspase-3, caspase-9 and cyt-c in bMECs and increased apoptosis of bMECs, although these effects were mitigated by treatment with either bacteriophage for 8 h.

**Conclusions:**

Bacteriophages CM8-1 and SJT-2 mitigated *K. pneumoniae*-induced inflammation in bMECs cultured in vitro. Therefore, the potential of these bacteriophages for treating mastitis in cows should be determined in clinical trials.

## Background

Bovine mastitis caused by infection with pathogenic microorganisms reduces milk production and quality, inflicting huge economic losses in the dairy industry [1, 2]. *Klebsiella pneumoniae*, a common environmental pathogen, causes mastitis in dairy cows [3, 4], accounting for 10 to 13% clinical mastitis, second to *E. coli* [5, 6]. Although antimicrobials are the most common therapy for bovine mastitis caused by *K. pneumoniae*, it often has single or multidrug resistance [4], with increasing risk of recurrence after treatment.

Bovine mammary epithelial cells (bMECs) are the first line of defense against invading pathogens that enter the udder through the streak canal, at the distal end of the bovine teat [4, 7]. Thus, bMECs have critical roles in both nonspecific and specific immune defenses against infection by pathogenic microorganisms [7]; furthermore, they can be infected by a variety of pathogenic microorganisms that damage these cells, leading to mastitis [8, 9]. Toll-like Receptor 4 (TLR4) and the NF-κB pathway are classic regulators of inflammation [10]. Lipopolysaccharides (LPS), the main component of the cell wall of gram-negative bacteria, can activate the TLR4-mediated NF-κB pathway to induce an inflammatory response [11]. Inhibiting or knocking out TLR4 down-regulated expression of inflammatory factors in cells, thereby suppressing inflammation [11, 12]. Although a timely and appropriate inflammatory response can effectively contain invading pathogens, an excessive inflammatory response can cause various degrees of cellular damage, including apoptosis, necrosis or pyroptosis [13, 14]. *K. pneumoniae* can multiply rapidly in bovine mammary tissues, causing a severe inflammatory response and tissue damage [4, 15]. In bovine mastitis induced by *K. pneumoniae*, infection of bMECs is an important component. Antibiotics are commonly used to treat bovine mastitis and consequently, drug resistance in gram-negative bacteria, including *K. pneumoniae*, is rapidly increasing [3, 16, 17]. Therefore, it is important to identify alternatives to antibiotics for treating bacterial infections, especially for bovine mastitis caused by *K. pneumoniae*.

Bacteriophages are small viruses, nearly ubiquitous in the natural environment [18]. Bacteriophage can lyse pathogens *in vivo* and *in vitro* without harmful effects on host cells and have been widely used in various fields [19, 20]. For example, 3 Myoviridae bacteriophages were used as adjunctive therapy and were safe and effective with severe *Staphylococcus aureu*s infections [21]. Furthermore, a bacteriophage “cocktail” was very successful in controlling bacterial wilt in tomatoes [22]. However, use of a bacteriophage for treatment of bovine mastitis caused by *K. pneumoniae* has apparently not been reported. Therefore, our objective was to investigate the role of bacteriophages for controlling *K. pneumoniae* in an *in vitro* infection model of bMECs.

## Results

### Transmission electron microscopy (TEM) of bacteriophages

**Fig. 1:**
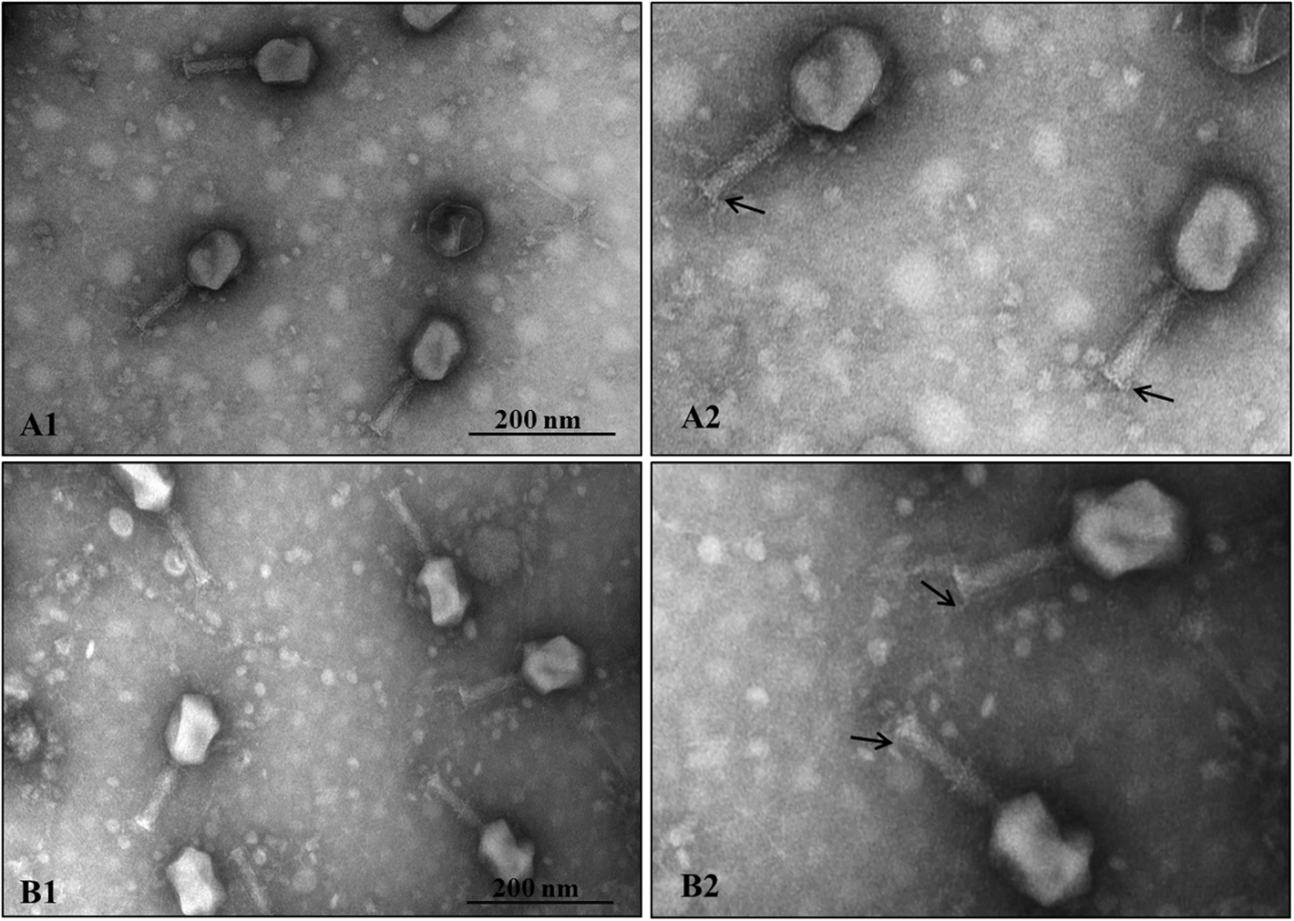
Transmission electron micrographs of *Klebsiella pneumoniae* bacteriophages CM8-1 and SJT-2. A1 and A2 are the ultrastructure of phage CM8-1, whereas B1 and B2 are the ultrastructure of phage SJT-2. Black arrows indicate tail filaments

Ultrastructures of *K. pneumoniae* bacteriophages CM8-1 and SJT-2 are shown (Fig. 1). Bacteriophages CM8-1 and SJT-2 were comprised of a head, neck and tail wire (Fig. 1A2, B2). Bacteriophages CM8-1 and SJT-2 had a polyhedral head, approximately 100 and 103 nm, respectively, whereas their tails were 103 and 116 nm and contained obvious tail filaments (Fig. 1A2, B2).

### Effect of bacteriophages on adhesion and invasion of *K. pneumoniae*

**Fig. 2:**
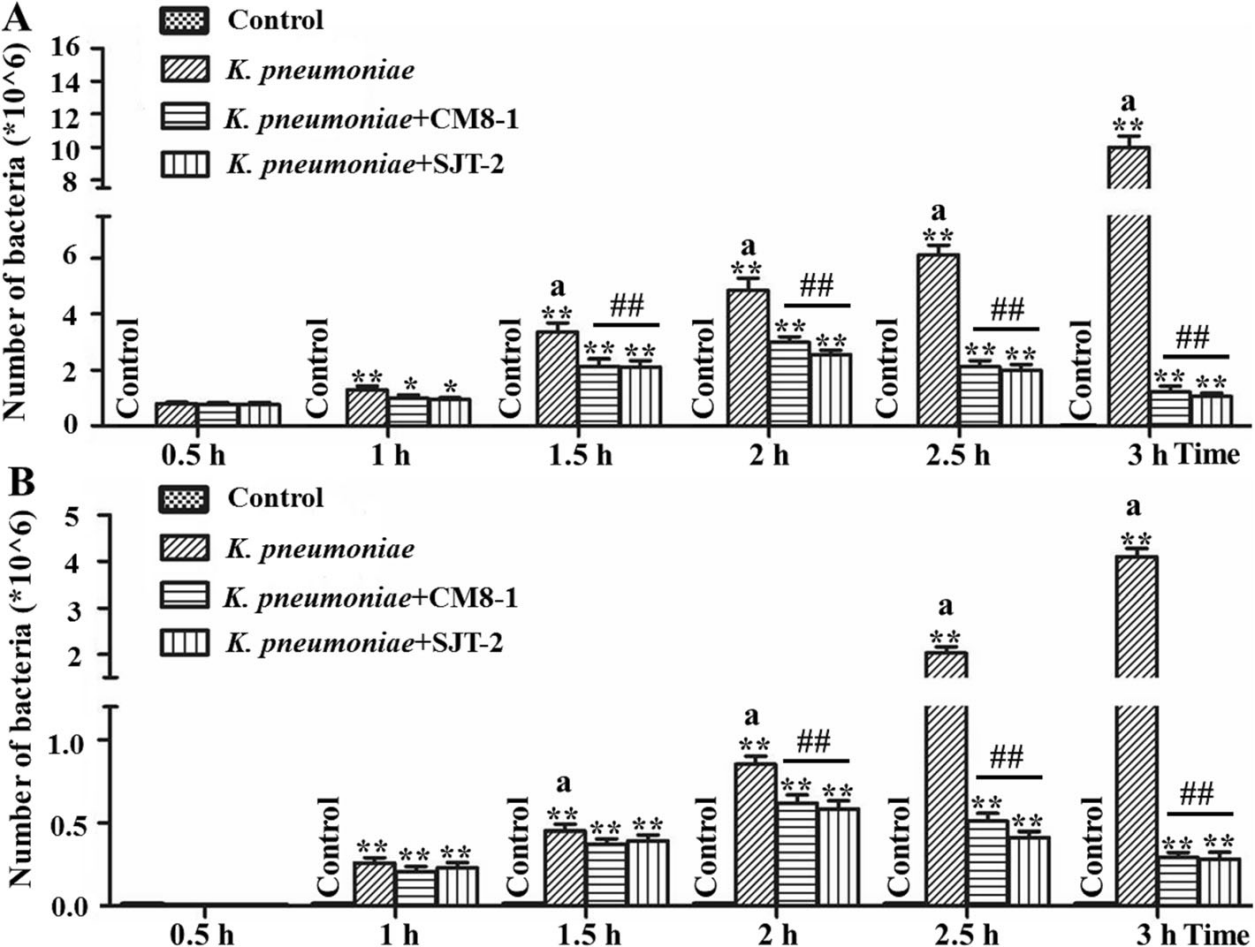
Adhesion and invasion of *Klebsiella pneumoniae*-infected bovine mammary epithelial cells in presence of bacteriophages CM8-1 or SJT-2. **a** Adhesion of *K. pneumoniae* to bMECs, **b** Invasion of *K. pneumoniae* into bMECs. Compared to the previous time point in the same group: ^a^*P* < 0.01; compared to the Control group: *P < 0.05, **P < 0.01; compared to the *K. pneumoniae* group: ^#^*P* < 0.05, ^##^*P* < 0.01

Adhesion of *K. pneumoniae* to bMECs in the *K. pneumoniae*, *K. pneumoniae* + CM8-1 and *K. pneumoniae* + SJT-2 groups were significantly increased in comparison to the Control (Fig. 2a). Starting soon after *K. pneumoniae* infection and as interval after infection increased, bacteriophages CM8-1 and SJT-2 reduced adhesion (*P* < 0.01) of *K. pneumoniae* to bMECs in the *K. pneumoniae* + CM8-1 and *K. pneumoniae* + SJT-2 groups as compared to the *K. pneumoniae* group at 1.5, 2, 2.5 and 3 h post infection (hpi) (Fig. 2a).

Adhesion of *K. pneumoniae* to bMECs in the *K. pneumoniae* + CM8-1 and *K. pneumoniae* + SJT-2 groups was similar to that in the *K. pneumoniae* group, without significant changes at 0.5 hpi (Fig. 2B). However, as duration of infection increased, adhesion of *K. pneumoniae* to bMECs in the *K. pneumoniae*, *K. pneumoniae* + CM8-1 and *K. pneumoniae* + SJT-2 groups increased (*P* < 0.01) compared to the Control group (Fig. 2B). Furthermore, adhesion of *K. pneumoniae* to bMECs in the *K. pneumoniae* + CM8-1 and *K. pneumoniae* + SJT-2 groups was lower (*P* < 0.01) than that of the *K. pneumoniae* group at 2, 2.5 and 3 hpi (Fig. 2B).

### Effect of bacteriophages on *K. pneumoniae-*induced cytotoxicity in bMECs

**Fig. 3:**
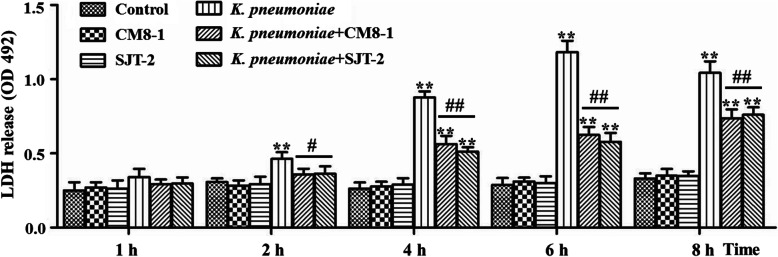
Lactate dehydrogenase (LDH) release from *Klebsiella pneumoniae*-infected bovine mammary epithelial cells in presence of bacteriophages CM8-1 or SJT-2. Compared to the Control group: *P < 0.05, **P < 0.01; compared to the *K. pneumoniae* group: ^#^*P* < 0.05, ^##^*P* < 0.01

There was no difference in LDH release among the Control, CM8-1 and SJT-2 groups (Fig. 3). At 2 hpi with *K. pneumoniae*, LDH release in the *K. pneumoniae* group was higher (*P* < 0.01) than in the Control group (Fig. 3), whereas at 4, 6 and 8 hpi, LDH release in the *K. pneumoniae*, *K. pneumoniae* + CM8-1 and *K. pneumoniae* + SJT-2 groups was greater (*P* < 0.01) than in the Control group (Fig. 3). At 2, 4, 6 and 8 hpi, LDH release in the *K. pneumoniae* + CM8-1 and *K. pneumoniae* + SJT-2 groups was significantly decreased compared to the *K. pneumoniae* group (Fig. 3).

**Fig. 4:**
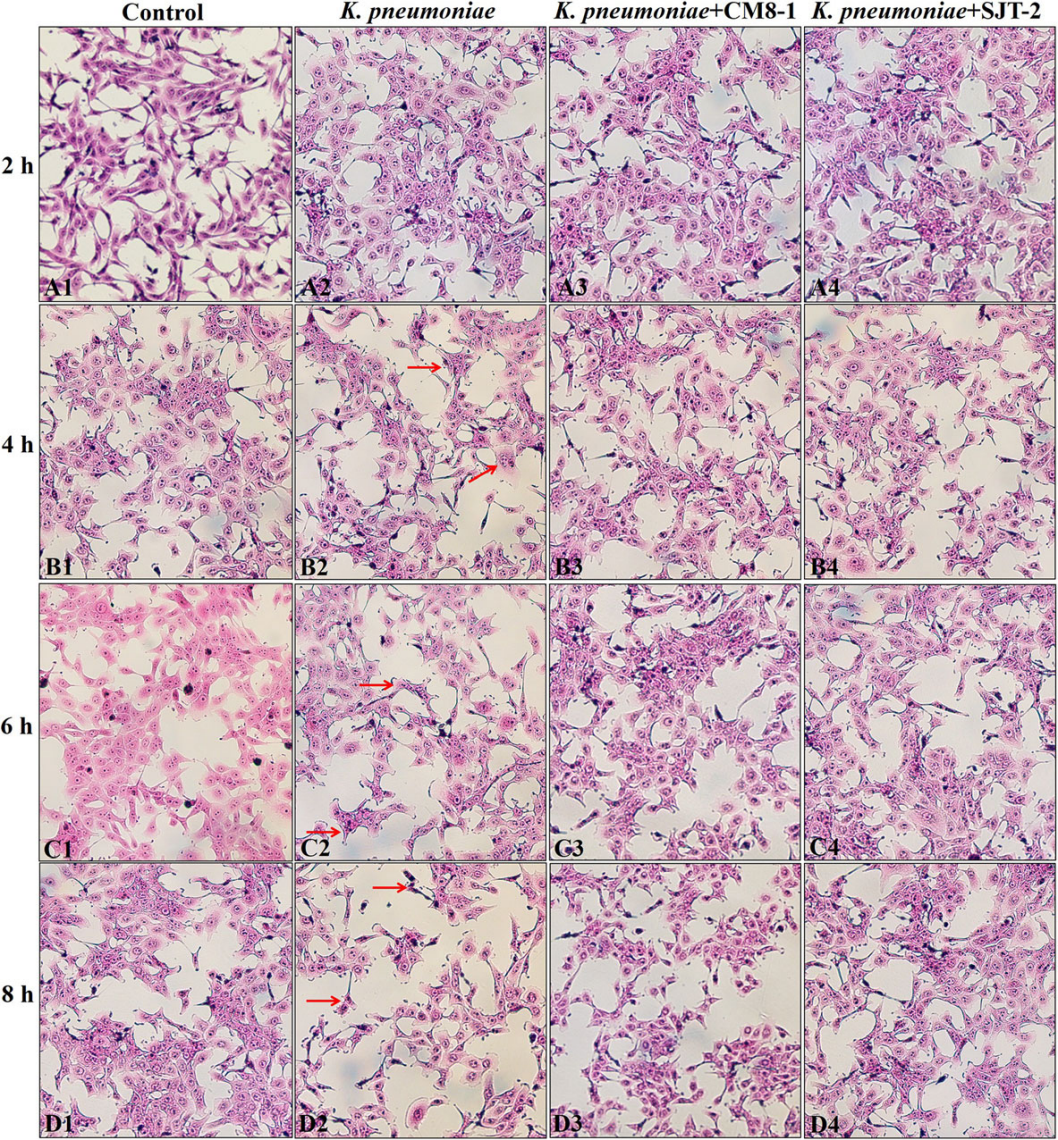
Morphological observations (40×) of *Klebsiella pneumoniae*-infected bMECs in presence of bacteriophages CM8-1 or SJT-2. A1-D1: Normal bMECs in the Control group at 2, 4, 6 and 8 h. A2-D2: Note damage to bMECs, including necrosis and swelling, shrinkage, loss of cell integrity, necrosis and heavy staining, and reductions in cell number at 2, 4, 6 and 8 h after infection in *K. pneumoniae* group. A3-D3 and A4-D4: Phages CM8-1 and SJT-2 mitigated cell damage caused by *K. pneumoniae* infection at 2, 4, 6 and 8 h in the *K. pneumoniae* + CM8-1 and *K. pneumoniae* + SJT-2 groups, respectively

### Morphological changes in bMECs

At 2 hpi with *K. pneumoniae*, morphological appearance was similar in the *K. pneumoniae* and Control groups. At 4 and 6 hpi with *K. pneumoniae*, there was no significant changes compared to the Control group (Fig. 4A1-C1, A2-C2). However, at 8 hpi with *K. pneumoniae*, damage to bMECs in *K. pneumoniae* group included cell swelling, nuclear hyperchromatism and necrosis, as well as reductions in number of bMECs, compared to the Control group (Fig. 4D1-D2). Furthermore, the extent of damage to bMECs gradually increased with interval after infection. However, morphology of bMECs in the *K. pneumoniae* + CM8-1 and *K. pneumoniae* + SJT-2 groups was similar compared to the *K. pneumoniae* and Control groups at 2 hpi. Swelling and necrosis of bMECs in the *K. pneumoniae* + CM8-1 and *K. pneumoniae* + SJT-2 groups were less severe compared to the *K. pneumoniae* group at 4, 6 and 8 hpi (Fig. 4A3-D3, A4-D4).

### Effect of bacteriophages on concentrations of inflammatory factors and inflammatory responses in *K. pneumoniae*-infected bMECs

**Fig. 5:**
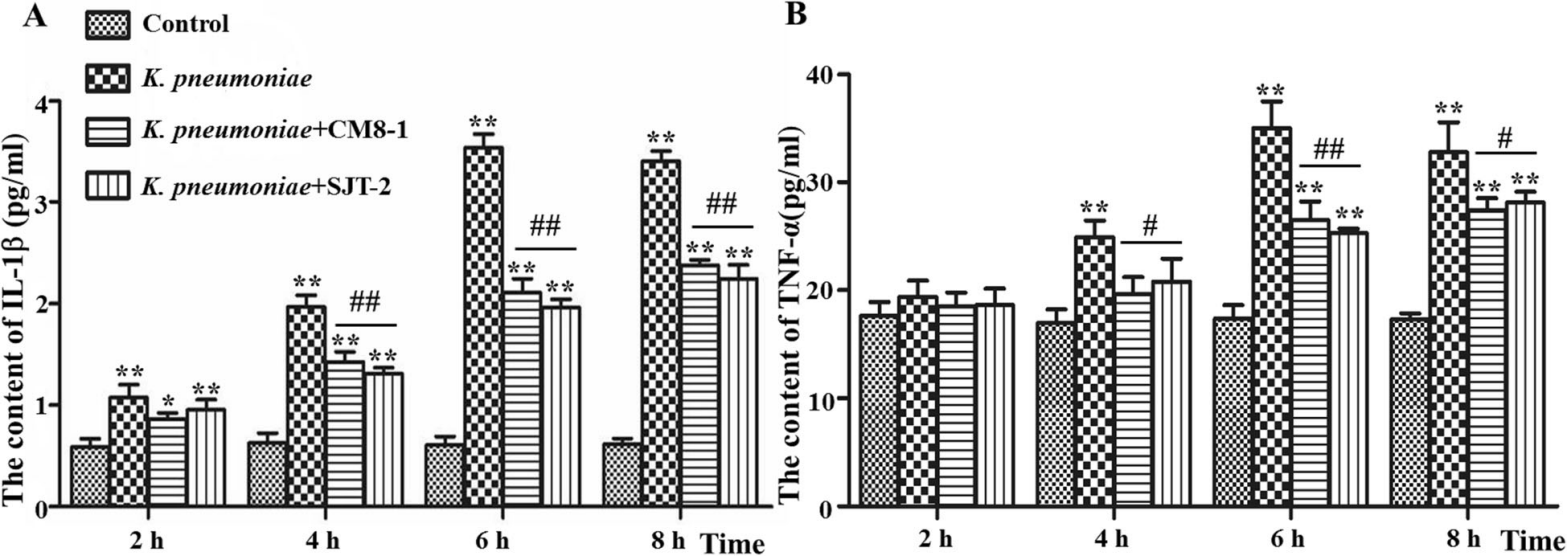
Concentrations of IL-1β and TNF-α proteins in the supernatant of *Klebsiella pneumoniae-*infected bovine mammary epithelial cells in presence of bacteriophages CM8-1 or SJT-2. **a** Content of IL-1β in the supernatant of *K. pneumoniae* infected bMECs, **b** Content of TNF-α in the supernatant of *K. pneumoniae* infected bMECs. Data presented as mean ± SD for 3 independent experiments. Compared to the Control group: *P < 0.05, **P < 0.01; compared to the *K. pneumoniae* group: ^#^*P* < 0.05, ^##^*P* < 0.01

Concentrations of IL-1β and TNF-α proteins were increased (*P* < 0.01) at 4, 6 and 8 hpi with *K. pneumoniae* (Fig. 5a and b). However, TNF-α and IL-1β concentrations in the *K. pneumoniae* + CM8-1 and *K. pneumoniae* + SJT-2 groups were significantly decreased in comparison to the *K. pneumoniae* group at 4, 6 and 8 hpi (Fig. 5a and b).

**Fig. 6:**
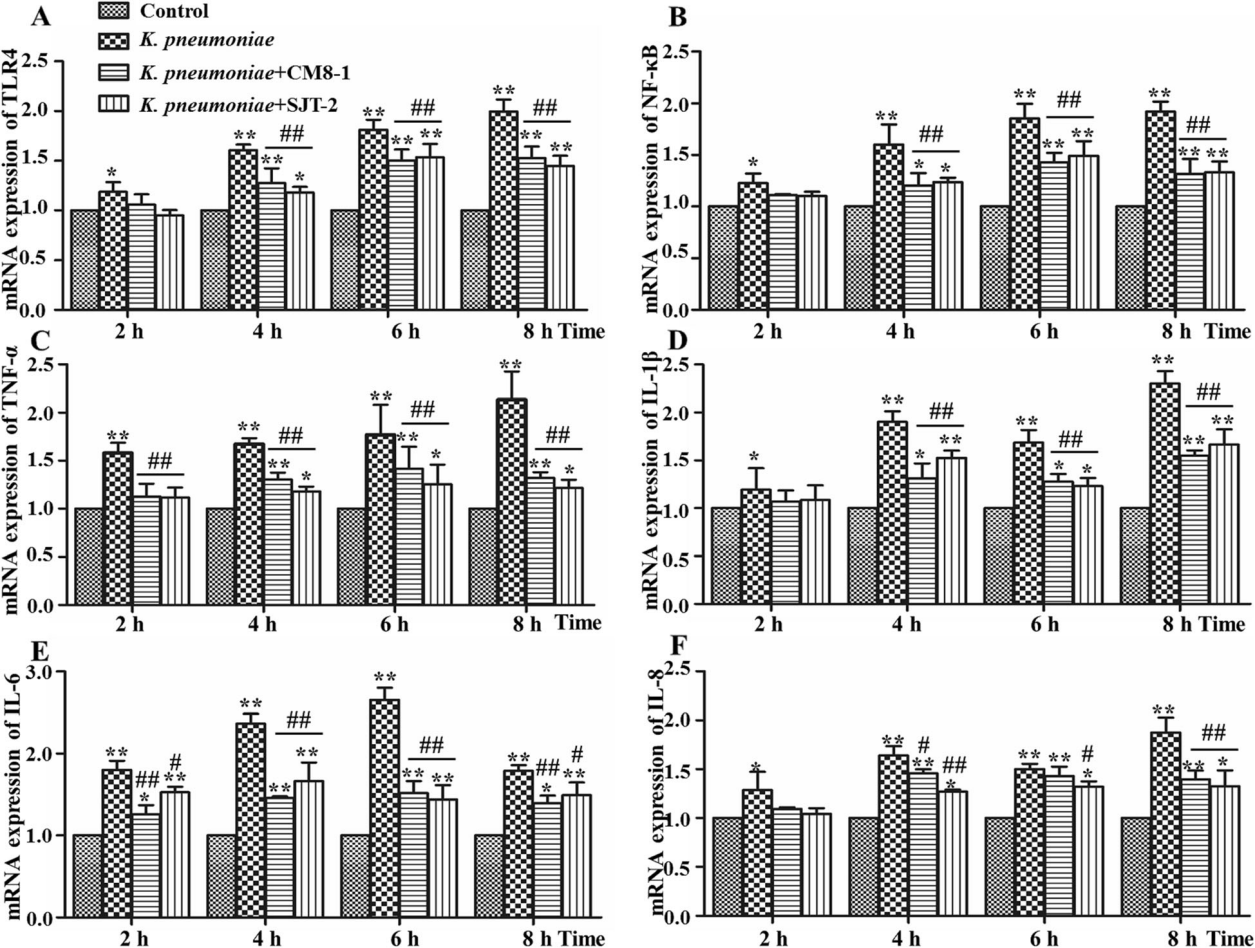
Relative mRNA expressions of TLR 4, NF-κB, IL-1β, TNF-α, IL-6 and IL-8 in *Klebsiella pneumonia* infected bovine mammary epithelial cells in presence of bacteriophages CM8-1 or SJT-2. Data presented as mean ± SD for 3 independent experiments. Compared to the Control group: *P < 0.05, **P < 0.01; compared to the *K. pneumoniae* group: ^#^*P* < 0.05, ^##^*P* < 0.01

Expression levels of TLR4, NF-κB and inflammatory factors (TNF-α, IL-1β, IL-6 and IL-8) in bMECs were determined by real-time PCR. At 2 hpi, mRNA expression levels of TLR4, NF-κB, TNF-α, IL-1β, IL-6 and IL-8 in bMECs were significantly higher in the *K. pneumoniae* versus Control groups (Fig. 6a-f). Furthermore, at 4, 6 and 8 hpi, mRNA expression levels of TLR4, NF-κB and inflammatory factors in the *K. pneumoniae*, *K. pneumoniae* + CM8-1 and *K. pneumoniae* + SJT-2 groups were significantly up-regulated compared to the Control group (Fig. 6a-f). In contrast, after treatment with bacteriophages CM8-1 and SJT-2, at 4, 6 and 8 hpi, mRNA expression levels of TLR4, NF-κB and inflammatory factors in the *K. pneumoniae* + CM8-1 and *K. pneumoniae* + SJT-2 groups were significantly down-regulated as compared to the *K. pneumoniae* group (Fig. 6a-f).

### Effect of bacteriophages on apoptosis in infected bMECs

**Fig. 7:**
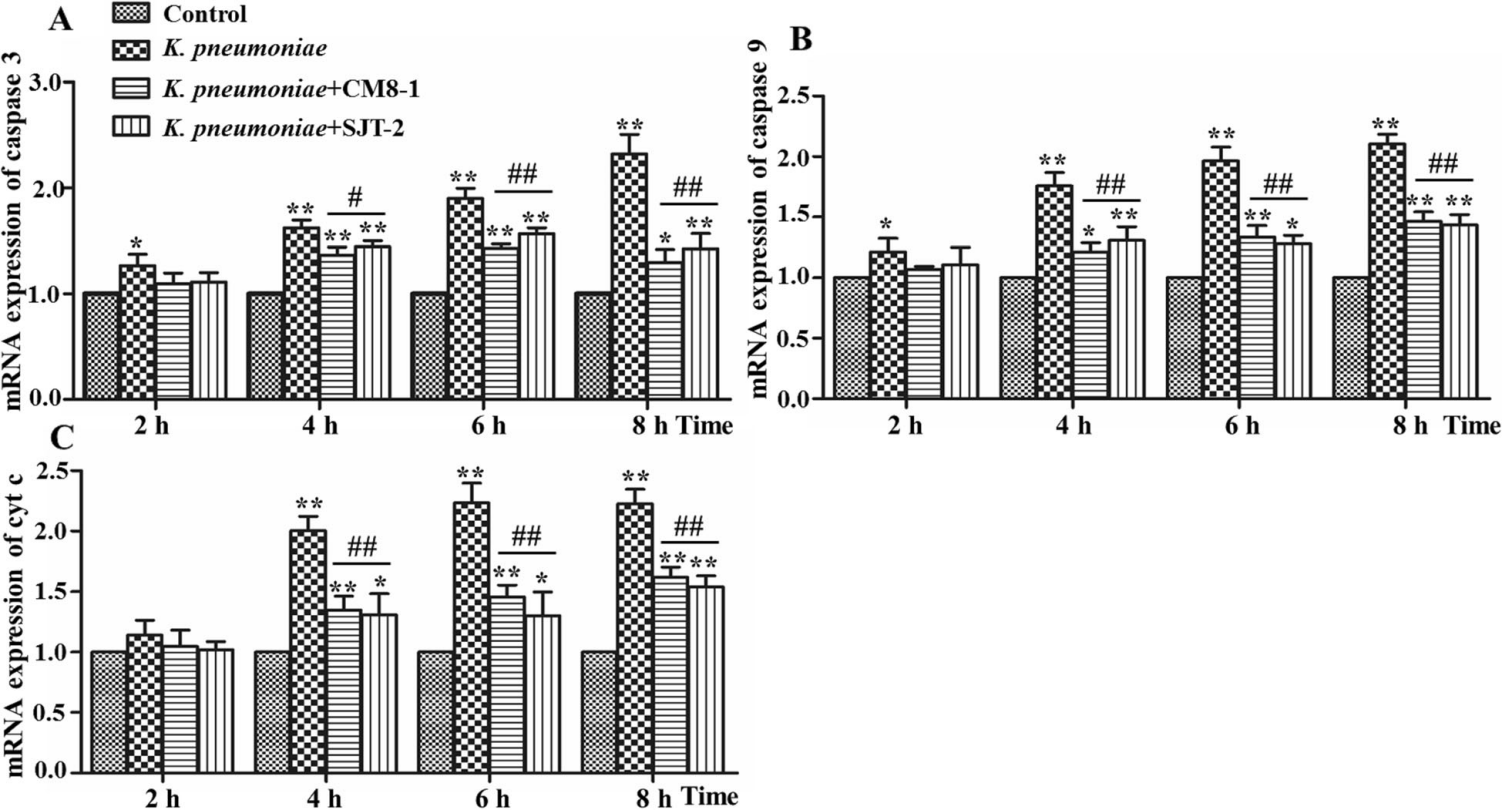
Relative mRNA expressions of apoptosis-related genes caspase 3, caspase 9 and cyt c in *Klebsiella pneumoniae*-infected bovine mammary epithelial cells in presence of bacteriophages CM8-1 or SJT-2. Data presented as mean ± SD for 3 independent experiments. Compared to the Control group: *P < 0.05, **P < 0.01; compared to the *K. pneumoniae* group: ^#^*P* < 0.05, ^##^*P* < 0.01

At 2 hpi of bMECs with *K. pneumoniae*, mRNA expression levels of apoptosis factors caspase-3 and caspase-9 were higher (*P* < 0.05) in the *K. pneumoniae* groups than in the Control group (Fig. 7a, b). At 4, 6 and 8 hpi, mRNA expression levels of apoptosis factors in the *K. pneumoniae*, *K. pneumoniae* + CM8-1 and *K. pneumoniae* + SJT-2 groups were significantly up-regulated compared to Controls (Fig. 7a-c). When compared to the *K. pneumoniae* group, at 4, 6 and 8 hpi, bacteriophages CM8-1 and SJT-2 significantly down-regulated mRNA expression levels of apoptosis factors in the *K. pneumoniae* + CM8-1 and *K. pneumoniae* + SJT-2 groups (Fig. 7a-c).

**Fig. 8:**
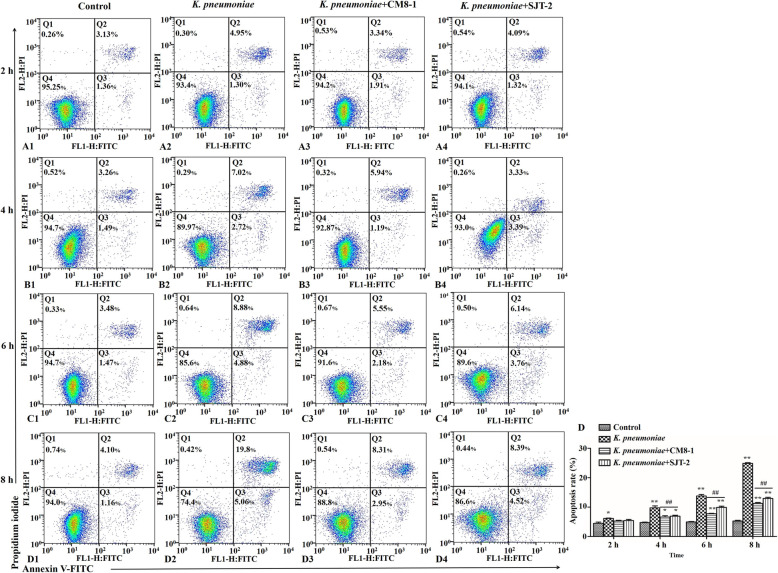
Flow cytometry to assess apoptosis rate by of *Klebsiella pneumoniae*-infected bovine mammary epithelial cells in presence of bacteriophages CM8-1 or SJT-2. A-D Percentage of apoptotic cells stained with FITC-conjugated annexin-V and PI (up to 8 hpi). E Percentage of apoptotic cells. Data are mean ± SD for 3 independent experiments. Compared to the Control group: *P < 0.05, **P < 0.01; compared to *K. pneumoniae* group: ^#^*P* < 0.05, ^##^*P* < 0.01

Apoptosis of bMECs in the *K. pneumoniae* group was higher (*P* < 0.05) than in the Control group at 2 hpi (Fig. 8). Furthermore, apoptosis rate of bMECs in the *K. pneumoniae* + CM8-1 and *K. pneumoniae* + SJT-2 groups was lower (*P* < 0.01) compared to the *K. pneumoniae* group at 4, 6 and 8 hpi (Fig. 8), although apoptosis was significantly higher in each of these groups compared to the Control group at all of these time points (Fig. 8).

## Discussion

This was apparently the first report that bacteriophages CM8-1 and SJT-2 isolated from mastitic bovine milk mitigated inflammatory responses and apoptosis in bMECs induced by *K. pneumoniae* by decreasing bacterial adhesion and invasion and suppressing expression of inflammatory factors. We concluded that bacteriophages CM8-1 and SJT-2 were protective in *K. pneumoniae*-infected bMECs and therefore have potential therapeutic value against bovine mastitis caused by *K. pneumoniae*.

*K. pneumoniae* can infect a variety of cells and tissues, inducing inflammatory responses and cell damage [23–25]. In addition, it also infects bMECs in development of mastitis in dairy cows [26]. When bacteria infect cells, bacterial adhesion and invasion are critical determinants of virulence [27]; therefore, the absence of genes related to synthesis of adhesion proteins reduce adhesion rates and cell toxicity. Furthermore, pathogens with high rates of adhesion and invasion have potential to cause serious damage to cells. In this study, adhesion and invasion of *K. pneumoniae* to bMECs increased significantly with interval after infection, although this was mitigated by bacteriophages CM8-1 and SJT-2. Bacteriophages can specifically lyse pathogens and therefore reduce adhesion and invasion. In the current study, bacteriophages CM8-1 and SJT-2 reduced ability of *K. pneumoniae* to infect and bMECs by mitigating adhesion and invasion.

Lactate dehydrogenase (LDH) is an important marker of bacterial cytotoxicity [28, 29]. Infection of bMECs with gram-negative bacteria, including *K. pneumoniae* and *E. coli*, increases LDH release and is accompanied by substantial cell damage [4, 30]. In this study, LDH significantly increased after bMECs were infected by *K. pneumoniae*, with increasing concentrations over time. Therefore, *K. pneumoniae* had toxic effects on bMECs, consistent with cell swelling and necrosis and decreasing numbers of bMECs.

A lack of toxic effects on host cells is characteristic of bacteriophages [19, 31]. Treatment of bMECs with either CM8-1 or SJT-2 bacteriophage did not increase release of LDH relative to the Control group, indicating that these bacteriophages were not toxic to bMECs. However, they can lyse and kill *K. pneumoniae*, reducing its virulence and mitigating damage to bMECs, manifested by less morphological damage and lower LDH concentrations.

Infection of bMECs by pathogens in development of bovine mastitis is usually accompanied by a severe inflammatory response [32]. Various pathogenic bacteria, including *K. pneumoniae, E. coli*, *S. aureus* and *S. agalactiae*, cause inflammation by producing inflammatory factors [4, 33–35]. In this study, concentrations of IL-1β and TNF-α in bMECs culture medium increased significantly after bMECs were infected with *K. pneumoniae*; furthermore, there was a positive association with duration of infection time, confirming *K. pneumoniae*-induced inflammation of bMECs. Gram negative bacteria such as *K. pneumoniae* and *E. coli* can activate TLR4 mediated NF-κB pathway to induce inflammation of bMECs [4, 11, 36, 37]. In this study, after exposing bMECs to *K. pneumoniae*, mRNA levels of TLR4 and NF-κB, in the pathway of regulating inflammatory response in bMECs, were significantly up-regulated and positively associated with duration of infection. In addition, downstream genes in TLR4 and NF-κB pathways, namely mRNA expression levels of TNF-α, IL-1β, IL-6 and IL-8 in bMECs were also up-regulated, confirming that *K. pneumoniae* induced an inflammatory response in bMECs. However, bacteriophages CM8-1 and SJT-2 were able to lyse *K. pneumoniae* and mitigate the inflammatory response induced by *K. pneumoniae*, manifested by decreased concentrations of IL-1β and TNF-α in bMECs culture medium, as well as reduced mRNA expression levels of TLR4, NF-κB, TNF-α, IL-1β, IL-6 and IL-8.

In this study, *K. pneumoniae* induced apoptosis of bMECs. Mitochondria are involved in the common apoptotic pathway, which mainly depends on the caspase families and cyt-c for regulation [38–40]. In the current study, *K. pneumoniae* infection of bMECs presumably enhanced release of cyt-c from mitochondria, triggered the caspase-9 and caspase-3 cascade reaction and accelerated cell apoptosis. The mRNA expression of caspase-3, caspase-9 and cyt-c in bMECs significantly increased with duration of infection time, implying apoptosis was triggered by *K. pneumoniae*. Based on flow cytometry, the apoptosis rate of bMECs increased gradually after *K. pneumoniae* treatment, which further confirmed that *K. pneumoniae* induced apoptosis of bMECs. However, bacteriophages CM8-1 and SJT-2 decreased expression of caspase-3, caspase-9 and cyt-c, as well as apoptosis rate of bMECs. Therefore, bacteriophages CM8-1 and SJT-2 mitigated apoptosis of bMECs induced by *K. pneumoniae*.

## Conclusions

Bacteriophages CM8-1 and SJT-2 mitigated *K. pneumoniae*-induced inflammation in bovine mammary epithelial cells cultured in vitro. Therefore, the potential of these bacteriophages for treating mastitis in cows should be determined in clinical trials.

## Methods

### Bacterial strains

The bacterial strain used in this study, isolated from clinical mastitis in a dairy cow, was *K. pneumoniae* (CM-KP8) with K57 capsule serotype [4]. This isolate was stored at the mastitis diagnostic laboratory, College of Veterinary Medicine, China Agricultural University. Prior to each experiment, fresh bacterial suspensions were prepared from frozen stocks by culture on LB nutrient agar medium and incubated at 37 ^o^C for 18 h. Thereafter, bacteria were sub-cultured on brain heart infusion broth for 18 h to mid-log phase and bacterial concentration was determined by the plate counting method for the following experiments.

### Bacteriophages

Bacteriophages CM8-1 and SJT-2 were isolated from dairy farm wastewater. Prior to each experiment, a bacterial solution in mid-log phase was mixed with bacteriophage stock solution and spread over a double-layer plate (96.6% LB liquid medium and 3.4% agar powder), with sodium magnesium (SM) buffer added after the bacteriophage had grown on the entire plate. Thereafter, this plate was placed on a shaker at 120 rpm/min for 2 h to liberate the bacteriophage from the plate and into the SM solution. The latter was collected and passed through a 0.22 µm filter to obtain the bacteriophage stock solution. Thereafter, PEG8000 (10%) was add into the bacteriophage suspension and left overnight at 4 ^o^C. Then, the bacteriophage suspension was centrifuged at 10,000 × g for 10 min to obtain bacteriophage precipitation. The above process was repeated twice to obtain pure bacteriophage. The bacteriophage precipitation was resuspended with DMEM for the following experiments.

### Cell culture and treatment

Bovine mammary epithelial cells (bMECs), line MAC-T (Shanghai Jingma Biological Technology Co., Ltd. China), were cultured in Dulbecco’s modified Eagle’s medium (DMEM) supplemented with 10% (v/v) Gibco® fetal bovine serum at 37 ^o^C in a humidified atmosphere of 5% CO_2_. Cells from 3 to 8 passages were used for experiments, with the same passages of cells used in each experiment. The bMECs were treated with or without *K. pneumoniae* (MOI, ratio of *K. pneumoniae* to bMECs was 10:1) and bacteriophages CM8-1 or SJT-2 (MOI, ratio of bacteriophage to *K. pneumoniae* was 1:10), to form the following groups: Control; *K. pneumoniae* infection group (*K. pneumoniae*), *K. pneumoniae* plus bacteriophage CM8-1 group (*K. pneumoniae* + CM8-1) and *K. pneumoniae* plus bacteriophage SJT-2 group (*K. pneumoniae* + SJT-2).

### Transmission electron microscopy (TEM)

A suspension (20 µL) of bacteriophages (titer = 10^9^ PFU/mL) was dropped on a copper mesh and precipitated for 2 min at room temperature, with excess liquid absorbed by filter paper. Bacteriophages were then stained with 2% uranium acetate for 1 min, excess liquid absorbed, naturally dried for 15 min at room temperature and then observed under a transmission electron microscope (H-7650, Hitachi, Tokyo, Japan) at 80 kV.

### Adhesion and invasion

Adhesion and invasion were measured as described [4]. The bMECs were inoculated into a 6-well cell culture plate and grown to 80% confluence, washed with phosphate buffer saline (PBS) and then DMEM supplemented with 1% (v/v) Gibco® Fetal Bovine Serum was added. At a multiplicity of infection (MOI, ratio of *K. pneumoniae* to cells) of 10:1, the bMECs were cultured at 37 °C in a humidified atmosphere of 5% CO_2_ for 0.5 h, and then with addition of bacteriophages CM8-1 or SJT-2 (MOI, ratio of bacteriophages to *K. pneumoniae*) of 1:10 for 0.5, 1, 1.5, 2 and 2.5 h. After incubation, cells were washed with PBS (to remove non-adherent bacteria), and the cell suspension was 10-fold diluted and cultured on LB liquid medium plate for counting colony forming units. Meanwhile, after incubation, cells were washed with PBS and treated with 1 mL of DMEM containing kanamycin (100 µg/mL) and then cultured at 37 °C in a humidified atmosphere of 5% CO_2_ for 2 h. The culture medium was removed, cells were lysed with 0.5% TritonX-100 and the lysate diluted and cultured on LB liquid medium plate as described above. Colonies were counted and invasion rate of *K. pneumoniae* into bMECs was calculated.

### Lactate dehydrogenase (LDH) assay

The bMECs were seeded in 96-well plates (density of 1 × 10^5^ cells/well), grown to 80% confluence, then DMEM supplemented with 1% (v/v) Gibco® Fetal Bovine Serum was added. Cells were then treated with *K. pneumoniae* and bacteriophages CM8-1 or SJT-2, as described above. After culture for 1, 2, 4, 6 or 8 h, supernatants were collected, centrifuged at 11,586 × g for 15 min at 4 ^o^C and LDH release measured by cytotoxicity (LDH Assay Kit-WST®, Dojingdo Laboratories, Kumamoto, Japan), according to manufacturer’s instructions. Absorbance of optical density was determined at 492 nm with a microplate reader (Bio-Rad, Hercules, CA, USA).

### Morphological observation of bMECs

The bMECs were seeded in 6-well plates with glass slides and grown to 80% confluence. Cells were then treated with *K. pneumoniae* and bacteriophages CM8-1 or SJT-2, as described above. Cells were cultured at 37 °C in a humidified atmosphere of 5% CO_2_ for 2, 4, 6 or 8 h. Then, cells were collected, fixed in 4% paraformaldehyde for 30 min, washed 3 times with PBS and dried naturally for 5 min. Hematoxylin-eosin (HE) staining was performed according to manufacturer’s instructions. An increasing alcohol gradient (75, 85, 95 and 100%) was used to dehydrate cells, which were subsequently observed under a light microscope at 40**×**.

### Detection of cytokine concentrations

Culture medium was collected, centrifuged at 11,586 × g for 10 min at 4 ^o^C and supernatant removed. Concentrations of inflammatory cytokines TNF-α and IL-1β in supernatant were measured using ELISA kits (Shanghai Zhenke Biotechnology Co., Ltd, Shanghai, China), used in accordance with manufacturer’s instructions.

### Quantifying cytokine mRNA with real‐time PCR

The bMECs were treated with *K. pneumoniae* and bacteriophages CM8-1 or SJT-2, and cells collected for total RNA extraction. Trizol Reagent (1 mL) was pre-chilled on ice and added to cell samples for 5 min to lyse cells. Mixed liquid was centrifuged at 11,586 × g for 15 min at 4 ^o^C and supernatant collected. Total mRNA of bMECs was extracted with mRNA extraction kit (TransGen Biotech Co., Ltd. Beijing, China), used according to manufacturer’s instructions. Primers for GAPDH (housekeeping gene), TLR4, NF-κB, TNF-α, IL-1β, IL-6, IL-8, caspase-3, caspase-9 and cyt-c were designed using primer 5.0 software (Table 1). Relative expression levels of TLR4, NF-κB, TNF-α, IL-1β, IL-6, IL-8, caspase-3, caspase-9 and cyt-c mRNA were determined using the Mx3000P™ RT-PCR system (Stratagene, USA) real-time PCR RG-3000A and the SYBR® Premix Ex Taq™ (Perfect Real Time) Kit (Takara, Japan).

**Table 1:** List of primers for real-time PCR^a^

Gene	Primer	Sequence (5’-3’)	Size (bp)
GAPDH	Forward Reverse	TCACCAACTGGGACGACA GCATACAGGGACAGCACA	206
TLR4	Forward Reverse	GGACCCTTGCGTACAGGTTG GGAAGCTGGAGAAGTTATGGC	155
NF-κB	Forward Reverse	GACCAAGGAGATGGACCTGA ACGATTTTCAGGTTGGATGC	150
TNF-α	Forward Reverse	ATGTGTGTGGAGAGCGTCAA GGGCCATACAGCTCCACAAA	145
IL-1β	Forward Reverse	ATGACTTCCAAGCTGGCTGTTG TTGATAAATTTGGGGTGGAAAG	114
IL-6	Forward Reverse	GCGCATCGGAGATGAATTGG AGATGGTCACTGTCCAACCAC	296
IL-8	Forward Reverse	AGTGCCTACGCACATGTCTTC TGCGTCACACAGAAACTCGTC	151
Caspase-3	Forward Reverse	GATGACATCGCCTGTCAGAA AATTCTGTTGCCACCTTTCG	203
Caspase-9	Forward Reverse	GTCACGGCTTTGATGGAGAT CAGGCCTGGATGAAGAAGAG	224
Cyt-c	Forward Reverse	TGCTGGTGATGTTGAGAAGG GTGTCCTCGTTCCAGGTGAT	327

^a^Annealing temperature for all primers was 60 °C

### Detection of apoptosis by flow cytometry

After bMECs were treated with *K. pneumoniae* and bacteriophages CM8-1 and SJT-2, apoptosis was assessed with an Annexin V-FITC apoptosis detection kit (Beyotime Biotechnology Co., Ltd, Shanghai, China). Cell culture fluid and washing fluid were collected. Then, cells were harvested with trypsin without EDTA and washed with PBS. Cells were centrifuged at 965 × g for 10 min, supernatant removed and cells re-suspended in 195 µL Annexin V-FITC binding solution and then transferred to a sterile flow cytometry glass tube. Annexin V-FITC (5 µL) was add to the cell suspension, followed by 10 µL propidium iodide staining solution. After 20 min at room temperature in dark conditions, apoptotic cells were detected using flow cytometry.

### Statistical analyses

Data were expressed as mean ± standard deviation (SD), followed by post hoc Tukey’s tests using SPSS 22.0 (SPSS Inc., Chicago, IL, USA). All measurements were replicated three times and *P* < 0.05 was considered significant.

## Data Availability

The datasets used and/or analyzed during the current study are available from the corresponding author on reasonable request.

## Abbreviations

*K. pneumoniae*: *Klebsiella pneumoniae*
LDH: Lactate dehydrogenase
bMECs: bovine mammary epithelial cells
MOI: Multiplicity of infection
TLR: Toll-like Receptor
SM: Sodium magnesium
DMEM: Dulbecco’s modified Eagle’s medium
HE: Hematoxylin-eosin
TEM: Transmission electron microscopy
PCR: Polymerase chain reaction
